# Transcriptomic insights into aerobic exercise-mediated attenuation of high-fat diet–induced muscle wasting

**DOI:** 10.3389/fphys.2026.1796142

**Published:** 2026-02-27

**Authors:** Weihao Hong, Jianrong Zheng, Yisheng Luan, Haibin Yu, Yueyun Xu, Bing Zhang

**Affiliations:** 1 School of Physical Education, Quanzhou Normal University, Quanzhou, China; 2 Division of Sports Science and Physical Education, Tsinghua University, Beijing, China; 3 School of Life Science and Technology, ShanghaiTech University, Shanghai, China

**Keywords:** aerobic exercise, high-fat diet, MICT, muscle wasting, transcriptome

## Abstract

Chronic consumption of high-fat diets (HFDs) induces obesity and metabolic dysfunction and is accompanied by progressive skeletal muscle wasting. Although aerobic exercise is generally considered less effective for maintaining muscle mass, accumulating evidence suggests that it can attenuate HFD-induced muscle wasting. However, the molecular mechanisms underlying this protective effect remain poorly defined. In this study, we investigated the effects of moderate-intensity continuous training (MICT) on HFD-induced muscle wasting and characterized the associated transcriptomic adaptations in the mouse gastrocnemius muscle. 21 weeks of HFD feeding increased body weight and serum glucose levels and induced marked muscle wasting, as evidenced by reduced gastrocnemius muscle index, impaired forelimb grip strength, decreased muscle fiber cross-sectional area, and excessive intramuscular lipid accumulation. These pathological alterations were significantly attenuated by an 8-week MICT intervention. RNA sequencing revealed that HFD predominantly induced a lipid-centered transcriptional program characterized by enhanced fatty acid uptake, trafficking, and β-oxidation. In contrast, MICT predominantly suppressed atrophy-associated genes (*Foxo1*, *Fbxo32*, and *Trim63*), while exerting minimal effects on myogenic genes (*Pax7*, *Myod1*, and *Myog*). Functional enrichment analyses further indicated that MICT modulated signaling pathways related to FoxO, PI3K–Akt, MAPK, and insulin signaling, together with biological processes associated with angiogenesis and calcium signaling. Collectively, these results suggest that MICT mitigates HFD-induced muscle wasting primarily by reprogramming the transcriptome from a lipotoxic, atrophic state toward a more insulin-sensitive, pro-angiogenic profile, with limited myogenic activation.

## 1 Introduction

The widespread adoption of high-fat diets (HFDs) in modern societies has contributed to a marked rise in obesity and related metabolic disorders (Abbasi and Khodadadi, 2025). Although the detrimental consequences of HFDs—such as adipose tissue expansion, chronic low-grade inflammation, and insulin resistance—are well documented, accumulating evidence indicates that skeletal muscle is also a direct target of diet-induced metabolic stress (He et al., 2020; Kawai et al., 2021; Kitessa and Abeywardena, 2016). Prolonged HFD consumption has been associated with decreased muscle mass, disorganization of myofiber structure, and impaired contractile function, changes that collectively compromise physical performance and metabolic health (Lee et al., 2015; Abrigo et al., 2016).

Resistance exercise is widely regarded as the most effective non-pharmacological strategy for preserving skeletal muscle mass. Its robust anabolic effects—classically mediated by activation of mechanistic target of rapamycin (mTOR) signaling, enhanced muscle protein synthesis, and increased expression of myogenic regulatory factors—have been consistently demonstrated in both clinical and experimental studies (Bodine et al., 2001; Fragala et al., 2019). In parallel, resistance training suppresses catabolic programs, including the ubiquitin–proteasome system and the autophagy–lysosome pathway, thereby shifting muscle toward an anabolic state (Kwon et al., 2018; Zanchi et al., 2009). These well-established adaptations underpin current recommendations that emphasize resistance training for the prevention and management of muscle-wasting conditions.

By contrast, aerobic (endurance) exercise has historically been viewed as less effective for preserving muscle mass, given that its canonical adaptations primarily involve improvements in cardiorespiratory fitness, mitochondrial biogenesis, and metabolic flexibility (An et al., 2024). Consequently, aerobic exercise has received comparatively limited attention in the context of muscle wasting. Nonetheless, emerging evidence indicates that aerobic exercise can attenuate muscle loss during metabolic stress, including that induced by HFD feeding (Xu et al., 2025; Zhang et al., 2021). However, the molecular mechanisms through which aerobic exercise mitigates HFD-induced muscle wasting remain insufficiently characterized. This gap is clinically relevant because aerobic exercise—particularly moderate-intensity continuous training (MICT)—is often more accessible and feasible than resistance training for individuals with joint limitations, severe obesity, or low baseline muscle strength. Defining the mechanisms underlying these benefits would therefore strengthen the biological rationale for considering aerobic exercise, especially MICT, as a practical strategy to manage obesity-associated muscle wasting.

To address this gap, we examined the effects of MICT on the gastrocnemius muscle in a mouse model of HFD-induced muscle wasting. RNA sequencing was performed to identify MICT-responsive transcriptional programs and signaling pathways associated with muscle preservation. By integrating physiological phenotyping with transcriptomic profiling, this study delineates the role of MICT in preserving skeletal muscle during HFD feeding and provides a mechanistic framework for its protective effects.

## 2 Materials and methods

### 2.1 Animals

3 week-old male C57BL/6J mice were obtained from the Tsinghua Laboratory Animal Resource Center. After 1 week of acclimation, mice were randomly allocated to four groups (n = 12 per group): standard chow diet (C), standard chow diet plus MICT (CM), HFD (H), and HFD plus MICT (HM). Standard chow diet (D12450J, containing 10 kcal% fat) and HFD (D12492, containing 60 kcal% fat) were procured from Research Diets. All animal procedures were approved by the Institutional Animal Care and Use Committee of Tsinghua University (Approval No. THU-LARC-2025–034) and were conducted in accordance with the ARRIVE guidelines.

### 2.2 Exercise protocol

The dietary intervention was maintained throughout the entire experiment (weeks 1–21). Starting at week 13, mice assigned to the exercise groups completed a 1-week treadmill acclimation phase (10 m/min for 10 min/day, 5 days/week), followed by an 8 week MICT intervention (weeks 14–21). Each session lasted 45 min and was performed 5 days per week, preceded by a 10-min warm-up at 10 m/min. Treadmill speed was progressively increased from 13 m/min (weeks 14–15) to 15 m/min (weeks 16–17), and then to 17 m/min (weeks 18–21), as previously described (Luan et al., 2025). These speeds corresponded to approximately 60% of the maximal oxygen consumption (VO_2_max) determined in HFD-fed mice (Supplementary Table S1).

### 2.3 Serum glucose measurements

Mice were fasted for 12 h and then anesthetized with Avertin (250 mg/kg, intraperitoneal injection). Blood was collected by retro-orbital bleeding, and the mice were subsequently euthanized by cervical dislocation. Serum was separated by centrifugation at 3,000 rpm for 15 min at 4 °C. Serum glucose levels were measured using an automated chemistry analyzer (Kehua ZY KHB1280).

### 2.4 Hematoxylin-eosin (HE) staining

The gastrocnemius muscle was dissected on ice, fixed in 4% paraformaldehyde, embedded in paraffin, and sectioned at 5 μm. The sections were then deparaffinized twice with xylene for 10 min each, dehydrated through a graded series of ethanol solutions (100%, 95%, 85%, 75%) for 3 min each, and rinsed in distilled water for 2 min. Staining was performed by immersing the sections in hematoxylin for 5 min, rinsing in water, differentiating in differentiation solution for 30 s, rinsing twice in tap water for 3 min each, and staining in eosin for 2 min. After staining, the sections were dehydrated through a graded series of ethanol solutions (75%, 85%, 95%, 100%) for 5 s each, washed in 100% ethanol for 1 min, cleared in xylene twice for 1 min each, and mounted with neutral resin. The sections were then observed and photographed under a microscope, and ImageJ software was used to measure the cross-sectional area of the muscle fibers.

### 2.5 Oil Red O staining

Following the dissection of the gastrocnemius muscle on ice, the tissue was frozen in liquid nitrogen and subsequently sectioned at 10 μm. Upon reaching room temperature, the sections were treated with staining buffer for 3 min. After the removal of excess buffer, the sections were incubated with Oil Red O staining solution at room temperature for 15 min. Subsequent to the removal of surplus staining solution, differentiation solution was applied for 5 min. The sections were rinsed in distilled water for 1 min, stained with hematoxylin for 2 min, rinsed again in distilled water for 1 min, and blued in water for 5 min. Finally, the sections were mounted for microscopic examination. ImageJ software was used to quantify the proportion of cells with lipid deposition in the gastrocnemius muscle.

### 2.6 Quantitative real-time PCR

Total RNA extraction was performed with TRIzol (Invitrogen) according to the manufacturer’s instructions. cDNA was synthesized using HiScript II Q RT SuperMix (Vazyme). Quantitative RT-PCR was performed using AceQ qPCR SYBR Green Master Mix (Vazyme).

The following primers were used:

*Gapdh* Forward: AGAAGGTGGTGAAGCAGGCATCT
Reverse: CGGCATCGAAGGTGGAAGAGTG
*Trim63* Forward: GTGTGAGGTGCCTACTTGCTC
Reverse: GCTCAGTCTTCTGTCCTTGGA
*Fbxo32* Forward: CAGCTTCGTGAGCGACCTC
Reverse: GGCAGTCGAGAAGTCCAGTC
*Foxo1* Forward: CCCAGGCCGGAGTTTAACC
Reverse: GTTGCTCATAAAGTCGGTGCT
*Pax7* Forward: TCTCCAAGATTCTGTGCCGAT
Reverse: CGGGGTTCTCTCTCTTATACTCC
*Myod1* Forward: CCACTCCGGGACATAGACTTG
Reverse: AAAAGCGCAGGTCTGGTGAG
*Myog* Forward: CTGTTTAAGACTCACCCTGAGAC
Reverse: GGTGCAACCATGCTTCTTCA

### 2.7 Gastrocnemius muscle RNA-seq

RNA-seq (n = 6 per group) was conducted by Biomarker (Beijing, China). Paired-end reads underwent quality assessment and trimming using Trim-galore (v.0.6.0). Subsequently, alignment of the reads to the mouse genome reference (GRCm38.p6) sourced from GENCODE was executed using STAR (v.2.7.3a). Following alignment, featureCounts (v.1.6.3) was employed to quantify the reads mapped to exon sites of genes listed in GTF files obtained from GENCODE. Differential expression analysis was performed utilizing DESeq2 (v.1.22.2), with raw counts utilized as input data. *P*-values were adjusted for multiple testing using the Benjamini–Hochberg method to control the false discovery rate (FDR), and genes with adjusted *P*-value (FDR) < 0.05 were considered significantly differentially expressed. Functional enrichment analyses, encompassing Kyoto Encyclopedia of Genes and Genomes (KEGG) and gene ontology (GO) analyses of differentially expressed genes (DEGs), were carried out using clusterProfiler (v3.10.1).

### 2.8 Statistical analysis

Statistical analyses were performed using GraphPad Prism 7 (GraphPad Software). Data were shown as mean ± SD. One-way analysis of variance (ANOVA) was employed for comparisons among multiple groups, followed by Tukey’s post-hoc test for pairwise com parisons. *P* < 0.05 was considered statistically significant.

## 3 Results

### 3.1 MICT attenuated HFD-induced obesity and muscle wasting

**FIGURE 1:**
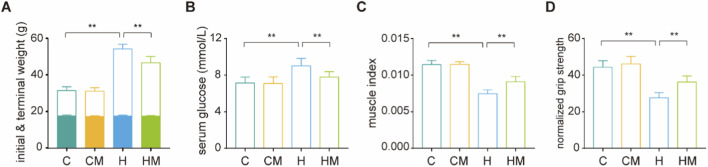
MICT attenuated HFD-induced obesity and muscle wasting. **(A)** Body weight, with the colored portion of each bar indicating initial body weight; **(B)** serum glucose levels; **(C)** gastrocnemius muscle index, defined as bilateral gastrocnemius muscle mass normalized to body weight; **(D)** normalized forelimb grip strength, defined as forelimb grip force normalized to body weight. **P < 0.01.

After 1 week of adaptive feeding, mice were assigned to four groups: standard chow diet (C), standard chow diet + MICT (CM), HFD (H), and HFD + MICT (HM). The initial body weights were comparable among all groups (Figure 1A).

**FIGURE 2:**
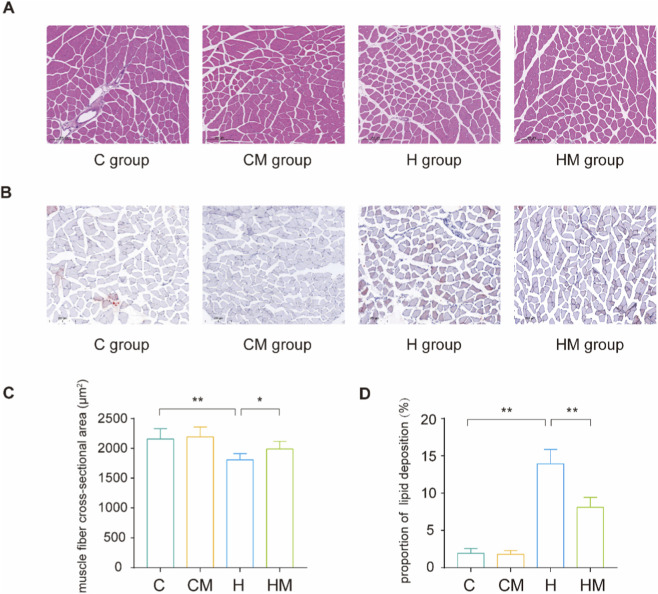
MICT mitigated HFD–induced muscle fiber atrophy and lipid accumulation. **(A)** HE staining and **(B)** Oil Red O staining results of the gastrocnemius muscle in the C, CM, H, HM groups; **(C)** statistical results of average muscle fiber cross-sectional area; **(D)** statistical results of lipid deposition ratio. The scale bar in A and B is 200 μm **P < 0.01, *P < 0.05.

At the experimental endpoint, body weight and serum glucose levels were significantly elevated in the H group compared with the C group, but were markedly reduced in the HM group relative to the H group (Figures 1A,B). Consistently, the gastrocnemius muscle index and normalized forelimb grip strength were significantly decreased in the H group and were significantly restored in the HM group (Figures 1C,D). Histological analysis further revealed reduced muscle fiber cross-sectional area, disrupted fiber organization, and increased lipid accumulation in the gastrocnemius muscle in the H group; in contrast, these pathological alterations were significantly attenuated in the HM group (Figures 2A–D).

### 3.2 Gastrocnemius muscle transcriptome analysis

**FIGURE 3:**
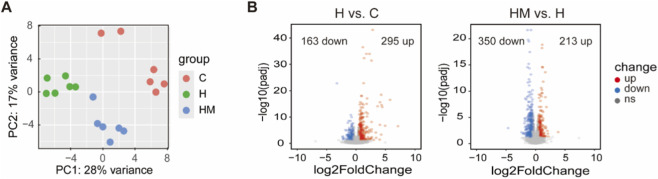
Gastrocnemius muscle transcriptome analysis. **(A)** PCA of gastrocnemius muscle transcriptome in C, H, HM groups. **(B)** Volcano plots of DEGs in H vs. C and HM vs. H.

We next performed RNA-seq to profile HFD-induced transcriptional changes and the effects of MICT intervention. PCA revealed distinct clustering of samples from the C, H, and HM groups, indicating clear group-level differences in global gene expression (Figure 3A). DEGs were identified using the criteria of an adjusted *P*-value <0.05 and |log2FC| ≥ 0.5. A total of 458 DEGs (295 upregulated, 163 downregulated) were identified between the C and H groups (H vs. C), and 563 DEGs (213 upregulated, 350 downregulated) were identified between the H and HM groups (HM vs. H) (Figure 3B). The top 50 significant DEGs in H vs. C were *BC048679, Decr1, Fabp3, Rab15, Ech1, Chrna2, Ephx2, Cyp1a1, Tph2, Dhrs4, Acadl, Ubd, Cd36, Itgad, Hadhb, Acot1, Slc35f5, Plin5, Mmp12, Oxtr, Slc5a7, Plbd1, Slc25a20, Eci2, Fbp2, Btnl9, Pex11a, Angptl4, Acaa2, Stau2, Slc5a3, Mlf1, Dbi, Lgr6, Hadha, Impa2, Acot2, Slc4a4, G0s2, Cpt2, Retsat, Rgcc, Slc25a34, Etfdh, Plin2, C7, Hsdl2, Npr3, Aifm2, Cryab* (Table 1; Supplementary Table S2). The top 50 significant DEGs in HM vs. H were *Angptl7, Txnip, Pkp4, Fbxo32, Sec14l5, Dach1, Fibin, Adh1, Galnt15, Tnfrsf19, Mrtfa, Inmt, Apold1, Klf11, Xdh, Cited4, Pdk4, Ptgfr, Relt, Ddit4l, Serinc3, Spock2, Slc43a3, Tnfaip2, Errfi1, Plcb4, Acvr1, Fmo2, Ret, Arhgap26, Fam110b, Nebl, Ky, Slx4ip, Fbxw7, Angptl4, Ucp3, Cry2, Lrrc38, Rora, Klhl38, Cd163, Mturn, Scara5, Trim63, Ptch1, Lvrn, Cd93, Car8, Abca6* (Table 2; Supplementary Table S3).

**TABLE 1:** The top 50 significant DEGs in H vs. C.

Gene symbol	log_2_ (foldchange)	Adjusted *P* value	Regulation
*BC048679*	2.810305	8.34E-44	Up
*Decr1*	1.137979	3.09E-37	Up
*Fabp3*	1.742903	1.04E-34	Up
*Rab15*	2.273352	1.04E-34	Up
*Ech1*	1.355624	4.32E-33	Up
*Chrna2*	2.621462	3.75E-32	Up
*Ephx2*	1.280299	1.43E-23	Up
*Cyp1a1*	−3.24577	1.55E-23	Down
*Tph2*	4.266208	3.36E-19	Up
*Dhrs4*	0.892765	7.81E-19	Up
*Acadl*	0.940393	1.00E-18	Up
*Ubd*	5.767995	2.70E-17	Up
*Cd36*	0.719191	2.06E-16	Up
*Itgad*	4.050096	2.16E-16	Up
*Hadhb*	1.005764	7.02E-16	Up
*Acot1*	1.78606	9.18E-16	Up
*Slc35f5*	0.635493	1.55E-15	Up
*Plin5*	1.230735	1.80E-15	Up
*Mmp12*	4.317651	2.46E-15	Up
*Oxtr*	3.65195	7.55E-15	Up
*Slc5a7*	4.444271	1.49E-14	Up
*Plbd1*	0.836759	1.90E-14	Up
*Slc25a20*	0.775148	1.74E-13	Up
*Eci2*	0.638861	5.37E-13	Up
*Fbp2*	0.909123	1.47E-12	Up
*Btnl9*	1.218367	4.01E-12	Up
*Pex11a*	0.953572	4.05E-12	Up
*Angptl4*	1.964385	4.38E-12	Up
*Acaa2*	0.925086	4.46E-12	Up
*Stau2*	0.532902	8.92E-12	Up
*Slc5a3*	0.843975	8.92E-12	Up
*Mlf1*	−0.89284	1.64E-11	Down
*Dbi*	0.802397	1.79E-11	Up
*Lgr6*	1.851906	6.18E-11	Up
*Hadha*	0.72542	8.21E-11	Up
*Impa2*	1.040845	9.41E-11	Up
*Acot2*	1.233048	9.41E-11	Up
*Slc4a4*	−0.76716	1.03E-10	Down
*G0s2*	1.119439	1.31E-10	Up
*Cpt2*	0.77179	1.58E-10	Up
*Retsat*	0.774455	2.13E-10	Up
*Rgcc*	1.012361	2.68E-10	Up
*Slc25a34*	1.017753	4.30E-10	Up
*Etfdh*	0.641324	5.72E-10	Up
*Plin2*	0.693641	7.56E-10	Up
*C7*	−1.25352	2.72E-09	Down
*Hsdl2*	0.693862	2.84E-09	Up
*Npr3*	1.327301	6.46E-09	Up
*Aifm2*	0.776045	1.36E-08	Up
*Cryab*	0.837016	1.57E-08	Up

**TABLE 2:** The top 50 significant DEGs in HM vs. H.

Gene symbol	log_2_ (foldchange)	Adjusted *P* value	Regulation
*Angptl7*	−1.35633	2.38E-22	Down
*Txnip*	−0.77591	2.38E-22	Down
*Pkp4*	−1.24768	1.61E-20	Down
*Fbxo32*	−1.13592	3.44E-20	Down
*Sec14l5*	1.126741	1.41E-19	Up
*Dach1*	1.080156	6.15E-19	Up
*Fibin*	−0.96892	3.49E-18	Down
*Adh1*	−1.15344	6.47E-16	Down
*Galnt15*	−1.35558	2.74E-15	Down
*Tnfrsf19*	1.147841	2.74E-15	Up
*Mrtfa*	1.18295	2.74E-15	Up
*Inmt*	−1.03643	4.52E-15	Down
*Apold1*	1.645677	2.45E-14	Up
*Klf11*	−0.79343	6.54E-14	Down
*Xdh*	−1.11045	5.42E-13	Down
*Cited4*	1.293297	1.53E-12	Up
*Pdk4*	−1.30562	2.21E-12	Down
*Ptgfr*	−1.06672	8.21E-12	Down
*Relt*	0.952813	2.47E-11	Up
*Ddit4l*	1.077754	5.57E-11	Up
*Serinc3*	−0.52489	6.73E-11	Down
*Spock2*	−1.16433	1.46E-10	Down
*Slc43a3*	−0.86852	1.46E-10	Down
*Tnfaip2*	1.039797	2.35E-10	Up
*Errfi1*	1.123973	2.35E-10	Up
*Plcb4*	−0.6351	3.68E-10	Down
*Acvr1*	0.599459	4.30E-10	Up
*Fmo2*	−0.97167	4.60E-10	Down
*Ret*	0.810938	5.63E-10	Up
*Arhgap26*	−1.22514	8.42E-10	Down
*Fam110b*	1.012301	9.78E-10	Up
*Nebl*	−1.40734	1.26E-09	Down
*Ky*	1.657281	1.32E-09	Up
*Slx4ip*	−0.77722	1.45E-09	Down
*Fbxw7*	0.598366	1.85E-09	Up
*Angptl4*	−1.74179	2.41E-09	Down
*Ucp3*	−1.55638	3.69E-09	Down
*Cry2*	−0.518	3.69E-09	Down
*Lrrc38*	0.824342	6.77E-09	Up
*Rora*	−0.6644	1.00E-08	Down
*Klhl38*	−1.16978	1.09E-08	Down
*Cd163*	−1.1075	1.09E-08	Down
*Mturn*	−0.67008	3.24E-08	Down
*Scara5*	−0.57302	3.24E-08	Down
*Trim63*	−1.28789	4.97E-08	Down
*Ptch1*	−0.67707	6.87E-08	Down
*Lvrn*	−1.1082	9.08E-08	Down
*Cd93*	0.619035	9.68E-08	Up
*Car8*	0.930244	9.73E-08	Up
*Abca6*	−0.91853	1.04E-07	Down

### 3.3 GO functional classification

**FIGURE 4:**
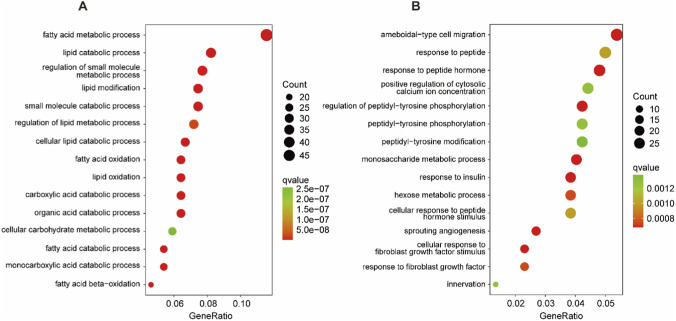
GO functional classification of the DEGs in **(A)** H vs. C and **(B)** HM vs. H.

To explore the biological functions of the DEGs, GO enrichment analysis was performed for the H vs. C and HM vs. H comparisons. In H vs. C, 184 terms were significantly enriched (q value <0.01). The top 15 significantly enriched terms were fatty acid metabolic process, fatty acid oxidation, lipid oxidation, lipid modification, fatty acid catabolic process, fatty acid beta-oxidation, monocarboxylic acid catabolic process, lipid catabolic process, cellular lipid catabolic process, carboxylic acid catabolic process, organic acid catabolic process, small molecule catabolic process, regulation of small molecule metabolic process, regulation of lipid metabolic process, cellular carbohydrate metabolic process (Figure 4A; Supplementary Table S4).

In HM vs. H, 60 terms were significantly enriched (q value <0.01). The top 15 significantly enriched terms (biological process) were regulation of peptidyl-tyrosine phosphorylation, response to peptide hormone, sprouting angiogenesis, response to insulin, ameboidal-type cell migration, cellular response to fibroblast growth factor stimulus, monosaccharide metabolic process, response to fibroblast growth factor, hexose metabolic process, cellular response to peptide hormone stimulus, response to peptide, positive regulation of cytosolic calcium ion concentration, innervation, peptidyl-tyrosine phosphorylation, peptidyl-tyrosine modification (Figure 4B; Supplementary Table S5).

### 3.4 KEGG pathway analysis

**FIGURE 5:**
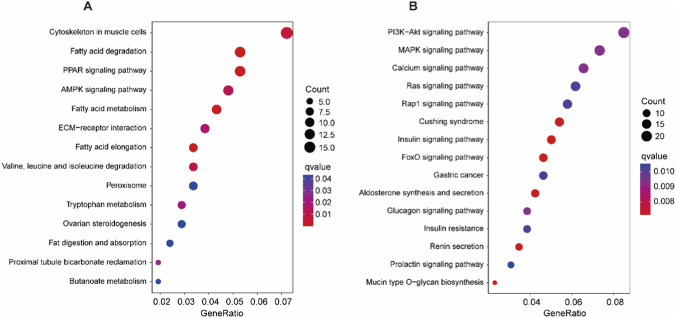
KEGG pathway analysis of the DEGs in **(A)** H vs. C and **(B)** HM vs. H.

To gain deeper insights into these DEGs, we further conducted KEGG pathway analysis. In H vs. C, DEGs were significantly enriched in 15 pathways (q value <0.05). They were fatty acid degradation, fatty acid elongation, PPAR signaling pathway, fatty acid metabolism, cytoskeleton in muscle cells, valine, leucine and isoleucine degradation, AMPK signaling pathway, ECM-receptor interaction, tryptophan metabolism, proximal tubule bicarbonate reclamation, fat digestion and absorption, ovarian steroidogenesis, butanoate metabolism, peroxisome, insulin signaling pathway (Figure 5A; Supplementary Table S6).

In HM vs. H, DEGs were significantly enriched in 32 pathways (q value <0.05). The top 15 significantly enriched pathways were aldosterone synthesis and secretion, insulin signaling pathway, Cushing syndrome, mucin type O-glycan biosynthesis, renin secretion, FoxO signaling pathway, PI3K-Akt signaling pathway, MAPK signaling pathway, glucagon signaling pathway, calcium signaling pathway, Ras signaling pathway, Rap1 signaling pathway, gastric cancer, insulin resistance, prolactin signaling pathway (Figure 5B; Supplementary Table S7).

### 3.5 Expression patterns of key myogenic and atrophy-related genes

**FIGURE 6:**
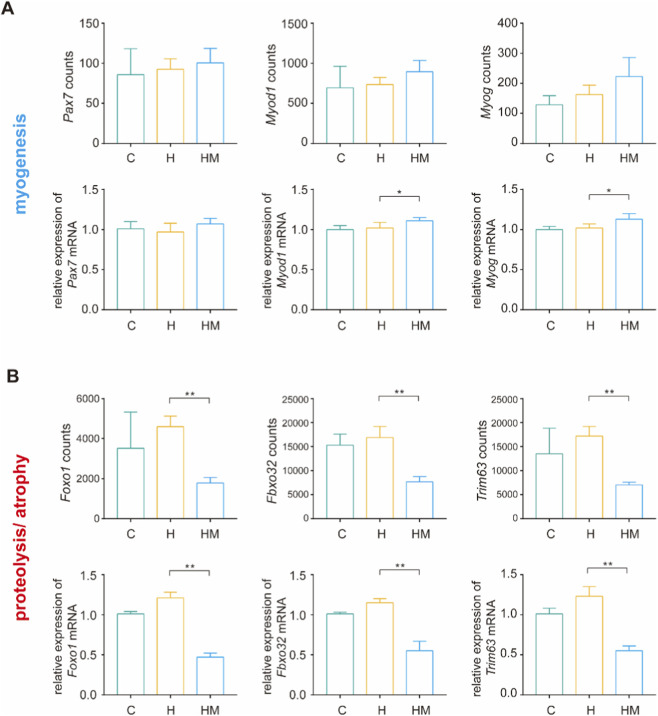
Expression patterns of key myogenic and atrophy-related genes. RNA-seq counts and relative mRNA expression levels of **(A)** myogenic genes and **(B)** proteolysis/atrophy genes. Relative mRNA expression was normalized to *Gapdh*. **P < 0.01, *P < 0.05.

As shown in Table 2, the top 50 significant DEGs in HM vs. H included multiple genes implicated in proteolysis and muscle atrophy, whereas genes indicative of myogenic activation were not present in this subset. Guided by this pattern, we selected representative markers of myogenesis (*Pax7*, *Myod1*, and *Myog*) and proteolysis/atrophy (*Foxo1*, *Fbxo32*, and *Trim63*) for validation (von Maltzahn et al., 2013; Bodine and Baehr, 2014; Tsuji et al., 2025; Adhikari et al., 2021). Compared with the H group, the HM group exhibited only modest changes in *Pax7*, *Myod1*, and *Myog* expression, whereas *Foxo1*, *Fbxo32*, and *Trim63* were markedly downregulated (Figures 6A,B). These trends were concordant between RNA-seq read counts and qPCR measurements, suggesting that the primary transcriptional effect of MICT is suppression of atrophy-associated programs, with limited induction of myogenic responses.

## 4 Discussion

In the present study, 21 weeks of HFD feeding caused a significant reduction in normalized grip strength, a lower gastrocnemius muscle index, decreased muscle fiber cross-sectional area, and excessive lipid accumulation, collectively indicating muscle wasting.

At the molecular level, these impairments were accompanied by pronounced transcriptional remodeling, characterized by strong induction of genes involved in fatty acid uptake and trafficking (*Cd36*, *Fabp3*, *Dbi*) and lipid droplet scaffolding (*Plin2*, *Plin5*), suggesting an increased capacity for fatty acid import, binding, and storage (Sztalryd and Brasaemle, 2017; Ramos-Jiménez et al., 2022; Li et al., 2022; Knudsen et al., 1993). Consistently, enrichment analyses highlighted GO terms related to fatty acid metabolism, lipid catabolic processes, and fatty acid β-oxidation, as well as KEGG pathways including fatty acid degradation, PPAR signaling, and fat digestion and absorption, pointing to a transcriptional state dominated by lipid handling.

HFD also elicited coordinated upregulation of mitochondrial and peroxisomal β-oxidation programs. Key transporters and enzymatic components, such as *Slc25a20* and *Cpt2*, together with multiple β-oxidation enzymes (*Acadl*, *Hadha*, *Hadhb*, *Acaa2*, *Ech1*, *Eci2*, *Decr1*, *Etfdh*), were significantly elevated, indicating a transcriptional shift toward enhanced fatty acid oxidation (Wanders et al., 2010; Wang et al., 2019; Tonazzi et al., 2021; Dissanayake et al., 2025). Consistent with this pattern, *Pex11a* and several fatty acid–modifying enzymes (*Acot1*, *Acot2*, *Retsat*, *Ephx2*, *Hsdl2*, *Dhrs4*) were also induced, paralleling enrichment of peroxisome-related and fatty acid elongation pathways. These features reflect an increased reliance on lipid utilization, a common adaptation to chronic lipid oversupply. While such remodeling may initially buffer nutrient overload, sustained activation of lipid oxidative pathways has been associated with the accumulation of reactive lipid intermediates, mitochondrial stress, and impaired insulin signaling, providing a plausible mechanistic link to insulin resistance (Aguer et al., 2015; Fazakerley et al., 2018; Muoio and Neufer, 2012).

Notably, enrichment of AMPK and insulin signaling pathways, together with altered expression of genes implicated in carbohydrate metabolism (*Fbp2*, *Slc5a3*, *Impa2*), suggests that carbohydrate handling and energy-sensing networks were engaged alongside the lipid-dominant shift (Bakshi et al., 2018; Brosch et al., 2022; Chatree et al., 2020). This pattern may indicate reduced metabolic flexibility, whereby skeletal muscle increasingly depends on fatty acid catabolism at the expense of efficient glucose utilization. Moreover, KEGG enrichment in valine, leucine and isoleucine degradation and butanoate metabolism suggests recruitment of branched-chain amino acid and short-chain fatty acid pathways, pointing to broader reprogramming of substrate oxidation under HFD feeding.

Beyond metabolic changes, transcriptomic signatures also suggested structural and microenvironmental remodeling. Enrichment of ECM–receptor interaction, together with upregulation of *Mmp12*, *Itgad*, and *Angptl4*, suggests activation of extracellular matrix turnover and low-grade inflammatory signaling, which may undermine contractile integrity (Alameddine and Morgan, 2016; Blythe et al., 2021; Zuo et al., 2023). Downregulation of *Slc4a4*, a bicarbonate transporter, may indicate reduced buffering capacity, potentially contributing to impaired contractile performance during repeated activity (Kurtz and Zhu, 2013).

Taken together, the transcriptomic profile of HFD-fed muscle reveals a lipid-centric metabolic state marked by coordinated upregulation of fatty acid uptake, storage, and oxidation pathways, accompanied by changes in carbohydrate handling, amino acid catabolism, and extracellular matrix remodeling. While these responses may be initially compensatory, they also establish a cellular milieu susceptible to lipotoxic stress, diminished metabolic flexibility, and compromised structural support, thereby providing a mechanistic framework for how chronic HFD predisposes skeletal muscle to dysfunction and insulin resistance.

Against the HFD-induced maladaptive changes described above, MICT markedly alleviated muscle wasting, as indicated by improvements in normalized grip strength, the gastrocnemius muscle index, fiber cross-sectional area, and lipid accumulation. At the molecular level, MICT reshaped the transcriptional landscape by suppressing genes associated with catabolic stress, atrophy, and metabolic inflexibility, while inducing genes linked to angiogenesis, insulin responsiveness, and structural remodeling, collectively reflecting exercise-mediated re-establishment of muscle homeostasis.

An important transcriptional signature of MICT was the repression of the FoxO-driven atrophy program. MICT decreased the expression of the transcription factor *Foxo1* and concomitantly downregulated its downstream E3 ubiquitin ligases *Trim63* and *Fbxo32*, key mediators of ubiquitin–proteasome–dependent proteolysis in muscle wasting (Peris-Moreno et al., 2020). In line with these gene-level changes, KEGG analysis showed significant enrichment of the FoxO signaling pathway, indicating that attenuation of FoxO-associated catabolic signaling is a major mechanism underlying MICT–mediated muscle preservation under HFD stress. In contrast, myogenic genes (*Pax7*, *Myod1*, *Myog*) exhibited only marginal alterations, indicating that MICT primarily reverses a pro-catabolic, wasting-associated transcriptional program rather than eliciting a robust pro-growth signature. In parallel, suppression of *Pdk4*—a key regulator that shifts substrate preference away from glucose oxidation toward fatty acid utilization—supports improved metabolic flexibility and potentially enhanced glucose handling (Pettersen et al., 2019). Reduced expression of *Txnip*, which is linked to oxidative stress and impaired insulin signaling, further suggests attenuation of redox stress and insulin-resistance pressure (Choi and Park, 2023). Likewise, downregulation of *Angptl4*, typically induced under lipotoxic and inflammatory conditions, is consistent with decreased intramuscular lipid burden.

In addition, MICT upregulated genes involved in cell survival, vascularization, and structural remodeling. For example, *Cited4*, a coactivator implicated in mitochondrial biogenesis and growth, was increased, potentially reflecting enhanced oxidative capacity (Lerchenmüller et al., 2020). *Mrtfa*, a regulator of actin dynamics and mechanosensitive transcription, together with *Apold1*, which mediates endothelial responses and angiogenic remodeling, points to vascular remodeling and support (Hyväri et al., 2020; Fan et al., 2023). In addition, the upregulation of *Acvr1* and *Ret* suggests broader engagement of growth factor and survival signaling cascades (Pacifici and Shore, 2016; Carlomagno et al., 2025). These findings align with GO enrichment in sprouting angiogenesis, response to insulin, and regulation of cytosolic calcium ion concentration, as well as KEGG enrichment in PI3K–Akt, MAPK, Rap1, and calcium signaling pathways, indicating transcriptional adaptations that may enhance insulin responsiveness, excitation–contraction coupling, and vascular supply, thereby supporting endurance capacity and functional resilience.

Nevertheless, several limitations should be acknowledged. First, the mechanistic inferences in this study are based on transcriptomic profiling of the gastrocnemius muscle. While RNA-seq provides a comprehensive view of gene regulation, it does not necessarily reflect protein abundance, post-translational modifications, enzymatic activity, or metabolic flux. Future studies integrating proteomics and metabolomics, together with targeted assays of pathway activity and substrate utilization, will be important to strengthen causal links between the observed transcriptional programs and functional outcomes. Second, we implemented an 8-week MICT intervention with progressive increases in treadmill speed; however, the present design does not allow determination of the optimal training dose. Systematic comparisons across training durations and intensities will be required to define a dose–response relationship for MICT in alleviating obesity-associated muscle wasting. Third, experiments were conducted exclusively in male mice; given well-documented sex differences in hormonal milieu, substrate metabolism, and exercise adaptation, inclusion of female cohorts will be necessary to improve generalizability.

## 5 Conclusion

These results suggest that MICT mitigates HFD-induced muscle wasting primarily by reprogramming the transcriptome from a lipotoxic, atrophic state toward a more insulin-sensitive, pro-angiogenic profile, with limited myogenic activation.

## Data Availability

The RNA-seq data are available in the Gene Expression Omnibus (GEO) database under accession number GSE319603.
