# Supplementary material for: Transcriptomic insights into aerobic exercise-mediated attenuation of high-fat diet–induced muscle wasting
Source: Front Physiol. 2026 Feb 27;17:1796142. doi: 10.3389/fphys.2026.1796142 (PMC12982024; doi:10.3389/fphys.2026.1796142)
Supplement: Supplementary file 4 [file Table1.docx]

**Supplementary Table 1. VO_2_max test**

| Training week | 1-2 | 3-4 | 5-6 | 7-8 |
| --- | --- | --- | --- | --- |
| VO_2_max（mL/h/kg） | 5780±137 | 6033±165 | 6260±118 | 6275±125 |
| VO_2_max（m/min） | 22 | 25 | 28 | 28 |
| 60%VO_2_max（m/min） | 13 | 15 | 17 | 17 |

The VO_2_max test began with an initial speed set at 3 m/min and a 0° incline, lasting for 5 minutes as a warm-up phase. Afterward, the speed was increased by 3 m/min every minute. During the incremental load exercise test, the criteria for reaching VO_2_max were met when any two of the following conditions were satisfied:

1) VO_2_ no longer increased with the increasing exercise load, forming a plateau or showing a change of no more than 5%;

2) the respiratory exchange ratio exceeded 1.0;

3) the mice reached exhaustion, demonstrated by the inability to keep up with the treadmill speed, contact of the abdomen with the treadmill surface, weakness in hindlimb push-off, and lack of response to electrical stimulation or manual encouragement.
